# New Insights into CRISPR-like Arrays in *Helicobacter pylori*: An Exploratory Analysis from Genomic Data

**DOI:** 10.3390/pathogens15050461

**Published:** 2026-04-24

**Authors:** Paloma Camacho-Aguilar, Javier Alejandro Delgado-Nungaray, Eire Reynaga-Delgado, Orfil Gonzalez-Reynoso, Libia Zulema Rodriguez-Anaya, Luis Alfonso Muñoz Miranda, Gabriel Rincón Enríquez, Inocencio Higuera-Ciapara, Luis Joel Figueroa-Yáñez

**Affiliations:** 1 Departamento de Biotecnología y Ciencias Alimentarias, Instituto Tecnológico de Sonora (ITSON), Ciudad Obregón 85000, Sonora, Mexico; paloma.camacho207086@potros.itson.edu.mx; 2 Chemical Engineering Department, University Center for Exact and Engineering Sciences, University of Guadalajara, Guadalajara 44430, Jalisco, Mexico; javier.dnungaray@alumnos.udg.mx (J.A.D.-N.); orfil.gonzalez@academicos.udg.mx (O.G.-R.); 3 Pharmacobiology Department, University Center for Exact and Engineering Sciences, University of Guadalajara, Guadalajara 44430, Jalisco, Mexico; eire.rdelgado@academicos.udg.mx; 4 SECIHTI-Instituto Tecnológico de Sonora, Ciudad Obregón 85000, Sonora, Mexico; libia.rodriguez@itson.edu.mx; 5 Centro de Investigación en Enfermedades Infectocontagiosas, Departamento de Microbiología y Patología, Centro Universitario de Ciencias de la Salud, Universidad de Guadalajara, Guadalajara 44340, Jalisco, Mexico; luis.mmiranda@academicos.udg.mx; 6 Laboratorio de Fitopatología, Biotecnología Vegetal, Centro de Investigación y Asistencia en Tecnología y Diseño del Estado de Jalisco A.C. (CIATEJ), El Bajío del Arenal, Zapopan 45019, Jalisco, Mexico; grincon@ciatej.mx; 7 Dirección de Investigación y Desarrollo, Universidad Anáhuac Mayab, Mérida 97308, Yucatán, Mexico; 8 Centro de Investigación en Alimentación y Desarrollo, A.C., Hermosillo 83304, Sonora, Mexico; 9 Unidad de Biotecnología Industrial, Centro de Investigación y Asistencia en Tecnología y Diseño del Estado de Jalisco A.C. (CIATEJ), Zapopan 45019, Jalisco, Mexico

**Keywords:** *Helicobacter pylori*, CagA pathogenicity island, CRISPR-like sequences, recombination hotspots, virulence gene stability

## Abstract

*Helicobacter pylori* (*H. pylori*) is a highly adaptable gastric pathogen with marked genomic plasticity. Whilst functional CRISPR-Cas systems provide adaptive immunity in many bacteria, they have not been identified in *H. pylori*, unlike CRISPR-like sequences. In this study, eight *H. pylori* genomes were analysed using the bioinformatics tools CRISPRCasFinder, CRISPRCasTyper, and CRISPRloci. A total of 25 CRISPR-like arrays were identified, showing high conservation (88%) both between and within strains, suggesting that these arrays are not random remnants but rather organised structures possibly involved in cellular processes. Notably, a structural association was observed between the CRISPR-like sequences and the cag pathogenicity island (CagA-PAI). Conversely, CagA-PAI instability in specific strains was observed in the presence of the TnpA and TnpB transposons. Furthermore, in strain 29CaP, CRISPR-like assemblies were located in genomic proximity to the prophage Helico 1961P, leading to the hypothesis of a compensatory or regulatory effect in the absence of CagA-PAI. Taken together, these findings indicate that CRISPR-like arrays in *H. pylori* characterise a genomic architecture within regions of high plasticity. This study provides a solid exploratory foundation for future functional research on the adaptive and pathogenic evolution of *H. pylori*.

## 1. Introduction

*Helicobacter pylori* (*H. pylori*) is a globally prevalent pathogen that colonises the gastric mucosa, infecting approximately 50% of the world’s population and representing one of the most common human infections. It is associated with various gastrointestinal pathophysiologies, such as peptic ulcers, gastritis, and gastric cancer [1]. Although not all people infected with this pathogen develop cancer, the Pan American Health Organization (PAHO)/World Health Organization (WHO) classifies *H. pylori* as a Group 1 carcinogen [2]. Thus, *H. pylori* is considered the most important infectious cause of cancer worldwide, responsible for ~80–90% of non-cardia gastric cancers.

Furthermore, *H. pylori* infection has been linked to an increased risk of neurological disorders, including Alzheimer’s and Parkinson’s diseases [3,4]. The growing resistance of *H. pylori* to commonly used antibiotics further complicates treatment strategies, posing a major global health challenge [5].

Among the most recent factors associated with antibiotic resistance in *H. pylori* is the *cagA* gene (cytotoxin-associated gene A), which is linked to heightened virulence in the gastric mucosa of infected hosts. The presence of *cagA*-positive strains has been associated with an increased risk of gastric cancer and peptic ulcer development [6].

The rising urgency for alternative therapeutic strategies against *H. pylori*, due to its notable antibiotic resistance, has led to the exploration of novel approaches to understand its pathogenicity [7]. Among these, the identification of Clustered Regularly Interspaced Short Palindromic Repeats (CRISPR) and CRISPR-associated (Cas) protein systems has emerged as one of the possible avenues, as CRISPR-Cas has revolutionised biological research as a genome-editing tool [8,9]. CRISPR-Cas systems are an adaptive immune mechanism found in almost 50% of bacteria and may influence the regulation of virulence factors with or without association with Cas proteins [10,11].

Although a functional CRISPR-Cas system has not yet been identified in *H. pylori*, CRISPR arrays have been reported, particularly in recent years, gaining relevance as they have been suggested to have biological implications [12]. These CRISPR-like sequences have been linked to the *vlpC* gene, homologous to *vacA* (a virulence factor in other microorganisms) [8,13], and correlated with more recent antibiotic resistance events [14]. In that sense, the search for functional systems and the characterisation of CRISPR arrays provide new data regarding the genomic architecture and the molecular determinants underlying its virulence.

Bioinformatics tools are key in the analysis and identification of CRISPR-Cas systems; combining them provides a more comprehensive understanding of these systems, as performance varies depending on the algorithm on which they are based. CRISPRloci, CRISPRCasTyper, and CRISPRCasFinder are among the most up-to-date tools for predicting CRISPR-Cas systems but also CRISPR arrays, in the case of CRISPRCasFinder, from the entire genome [15,16]. Additionally, visualisation tools, such as Proksee, facilitate the analysis of the genomic context, allowing the identification of neighbouring genes potentially involved in virulence or antibiotic resistance. In addition, some biotechnical control strategies could be detected, such as the use of bacteriophages or some activity related to them [17].

Therefore, the objective of this study was to analyse the presence of both CRISPR-Cas systems and CRISPR-like arrays in *H. pylori* strains by applying bioinformatic tools with particular emphasis on their genomic context to provide insights into their potential association with virulence-related elements.

## 2. Materials and Methods

### 2.1. Genome Data Collection

Eight genomes of *H. pylori* strains (VN1291, CHC155, SHIM-010, F57, HE93/10_v1, 24-A-EK1, 29CaP, and 7C) were retrieved from the National Centre for Biotechnology Information (NCBI) (https://www.ncbi.nlm.nih.gov/datasets/genome; accessed on 23 April 2025) (Table 1). The *H. pylori* strains were selected based on the availability of complete genomes from isolates from diverse geographic regions of the world and different gastrointestinal pathophysiologies, providing a diverse exploratory framework and detailed manual inspection of genomic regions.

### 2.2. CRISPR Array Identification

The identification and comparison of CRISPR-Cas systems and CRISPR-like arrays across the eight *H. pylori* genomes were performed using the bioinformatic tools: CRISPRCasFinder v4.2.20 [16], CRISPRCasTyper v1.8.0 [24] and CRISPRloci v5.0.12 [25]. This multi-tool bioinformatic approach was employed to reduce individual biases and to ensure that the detection (or absence) of these systems was not an artefact of a single algorithm. All analyses were conducted using default parameters, which represent validated benchmark settings for these tools across diverse bacterial species.

### 2.3. Validation of Bioinformatic Tools for CRISPR Array Identification

To validate the accuracy of the aforementioned bioinformatic tools, we used *H. mustelae* strain 12198 (NC_013949.1) as a positive control, as it is phylogenetically closely related to *H. pylori* within the *Helicobacter* genus that possesses a well-characterised CRISPR-Cas subtype II-C system and comparable pathogenic features [13,26,27]. To evaluate the structural characteristics and potential functionality of the CRISPR arrays, the direct repeat (DR) length and the number of spacers within each locus were analysed. Spacers were further searched against NCBI BLAST 2.17.0 databases (E-value: 10^−5^) to identify homology with prophage or plasmid sequences (https://blast.ncbi.nlm.nih.gov/Blast.cgi; accessed on 23 April 2025). These sequences were also searched in CRISPRCasdb [28].

### 2.4. Evaluation of Associated and Neighbour Proteins of CRISPR Arrays

The genomic neighbourhoods of CRISPR arrays were predicted and evaluated for neighbour and associated proteins in the genomes of *H. pylori* strains using CRISPRloci v5.0.12 [25] by applying the default parameters for all analyses.

### 2.5. Detection of Prophage Sequences

The prophage sequences integrated into the genomes of *H. pylori* were identified using PHASTEST v3.0 [29], using the same genome data described above.

### 2.6. Genomic Characterisation and Visualisation

For genomic context analysis, all *H. pylori* genomes analysed were re-annotated using Bakta v1.1.0 [30] to standardise the bacterial genome annotation. Events of horizontal gene transfer (HGT) and mobile genetic elements (MGEs) were mapped using Alien Hunter v1.3.0 [31] and mobileOGdb v1.1.3 [32], respectively. The integrated results were visualised as genomic maps using Proksee v1.0.0a6 [17], highlighting the location of the identified CRISPR arrays, as well as the detected prophage sequences.

## 3. Results

### 3.1. General Characteristics of the CRISPR-like Sequences Identified in H. pylori

**Figure 1:**
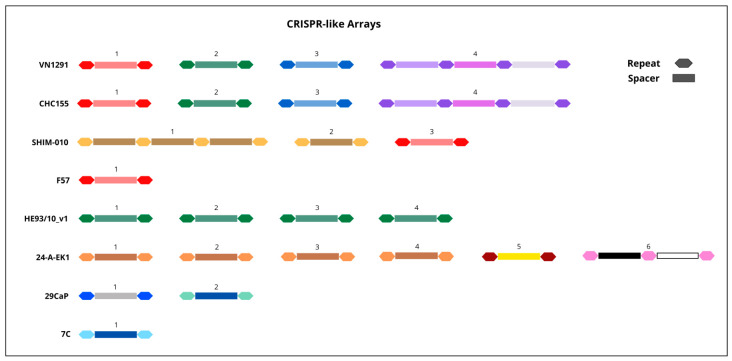
CRISPR-like arrays identified with CRISPRCasFinder and their characteristics. The colour pattern in the ‘DR consensus and Spacers’ indicates the homology between the respective sequences of the different arrays identified. Additional details are available in Table S1.

Bioinformatic analysis of the eight *H. pylori* genomes revealed a total of 25 CRISPR arrays comprising 33 spacers. Sequence homology was observed both within and between strains in 88% (22/25) of the identified CRISPR-like arrays (Figure 1). These elements were detected using CRISPRCasFinder, with an evidence level of 1. In contrast, CRISPRCasTyper and CRISPRloci did not identify CRISPR arrays in any of the strains analysed. Furthermore, none of the genomes harboured complete CRISPR-Cas systems or associated Cas proteins, which, together with the low evidence level, suggests the absence of functional interference machinery. Notably, all detected CRISPR-like arrays were located within HTG regions, as predicted by Alien Hunter v1.3.0 software.

### 3.2. Validation of CRISPR-Cas System and Array Analysis

The well-characterised CRISPR-Cas subtype II-C of *H. mustelae* 12198, composed of Cas1, Cas2, and Cas9 proteins, was correctly and completely identified with CRISPRCasFinder and CRISPRCasTyper. CRISPRloci also accurately identified the neighbouring proteins associated with this CRISPR-Cas system. In addition, CRISPRCasFinder enabled the detection of six CRISPR-like arrays (Table 2).

Compared with the functional CRISPR-Cas subtype II-C system of *H. mustelae* (Table S2), the DR sequences detected in the genomes of *H. pylori* ranged from 23 bp to 50 bp in length, whilst the spacers ranged from 1 to 3 sequences per CRISPR-like array, with lengths from 19 bp to 60 bp (Table S3).

Spacer homology searches in the CRISPRCasdb database found no matches with exogenous material, and BLAST analyses produced no significant alignments with known phages. However, certain spacers in the CRISPR-like arrays of strains HE93/10_v1 and 24-A-EK1 were found to be identical at different genomic locations (Table S1). This pattern reveals internal duplication of spacer sequences within the genome, although CRISPRCasFinder identified them as part of separate arrays. Given the absence of a functional cas operon, these repeated spacers could be recombination events or genomic rearrangements. The duplicated spacer sequences are presented below:

HE93/10_v1: AATAAAAAGAAAACAGGACAAGTAGCTAGCCCTG
24-A-EK1: GATCGGTTAGCCCTGAACCCATTTATGCTACGATTGATGATCTCGGCGGACCT

### 3.3. Associated and Neighbour Proteins of CRISPR Arrays

The presence of proteins associated with CRISPR-Cas systems was detected with CRISPRloci. These were located adjacent to five of the CRISPR-like arrays identified with CRISPRCasFinder in the VN1291, 24-A-EK1, and 29Cap strains of *H. pylori*. In VN1291, Cas5 and Cas10 were identified, additionally with an unknown protein approximately 20 kb upstream of CRISPR-like array No. 2. In the 24-A-EK1 strain, 10 kb upstream of CRISPR-like array No. 5 were identified presumptive Cmr5 and Cas8 proteins, the latter also located 40 kb upstream of CRISPR-like array No. 6. In the case of 29Cap strain, putative Cas12 and Csa5 proteins were located 30 kb upstream of the CRISPR-like array No. 1 and 2 kb adjacent to the CRISPR-like array No. 2. Details of the associated proteins can be found in Table S4.

### 3.4. Integrated Prophages Identified in H. pylori Genomes

Four distinct intact prophages were identified in the genomes of four *H. pylori* strains (Table 3). These regions exhibited mosaic composition, consisting of homologous genes to various phages of the *Helicobacter* genus, including Helico_1961P, KHP30, KHP40, and phiHP33. The prophage organisation corresponded to modular arrangements typical of the *Myoviridae* family.

**Figure 2:**
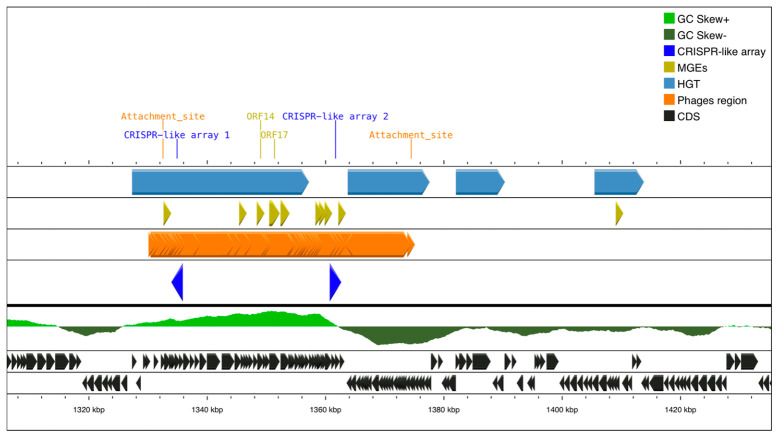
Helico 1961P prophage detected in the genome of *H. pylori* 29CaP. The CRISPR-like arrays identified in the strain are located overlapping the same genomic region as the prophage, suggesting a possible relationship between these elements. CDS refers to coding DNA sequences in the genome.

Throughout the phage region of the SHIM-010 strain, multiple attL and attR sites were detected, indicating potential recombination sites (Table S5). On the other hand, a single *attL* and *attR* site were detected in strains CHC155 (*attL*, 756,907 bp; *attR*, 791,804 bp), VN1291 (*attL*, 757,661 bp; *attR*, 784,469 bp), and 29CaP (*attL*, 1,332,515 bp; *attR*, 1,363,762 bp). In CHC155, SHIM-010, and VN1291 genomes, the prophage region was located between CRISPR-like array Nos. 2 and 3. Additionally, analysis of the genomic context of strain 29CaP revealed the overlap of the CRISPR-like arrays present in this strain with the prophage region (Figure 2).

### 3.5. CRISPR-like Arrays Associated with Pathogenicity Islands

Genomic context characterisation and visualisation revealed the presence of complete islands of pathogenicity associated with the CagA cytotoxin (CagA-PAI) in *H. pylori* strains CHC155, HE93/10_v1, and VN1291, as well as two fragmented CagA-PAIs in the genomes of strains 24-A-EK1 and SHIM-010. All pathogenicity islands involved are associated with HGT events.

**Figure 3:**
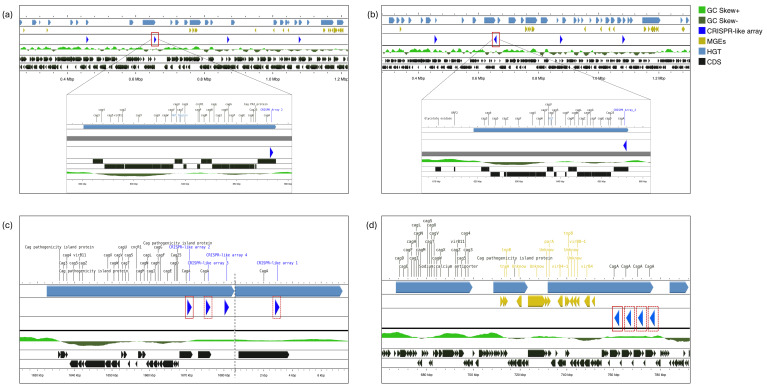
CagA-PAI is associated with CRISPR-like arrays identified in *H. pylori* genomes. (**a**) Strain VN1291, which presents a complete CagA-PAI located around 0.7 Mbp, adjacent to CRISPR-like array No. 2; (**b**) Strain CHC155, CRISPR-like array No. 2 is located similarly to strain VN1291. (**c**) Strain HE93/10_v1, CRISPR-like array No. 2 adjacent to the complete CagA-PAI. CRISPR-like arrays Nos. 1 and 3, marked with a dashed outline box, are associated with copies of *cagA* genes; (**d**) Strain 24-A-EK1, CRISPR-like array No. 1 marked with a solid line box adjacent to a fragmented CagA-PAI due to the possible integration of MGEs, including transposases (TnpA, TnpB), is shown in the central panel. 70 kb to the left of the CRISPR-like sequence, the genes and components of the T4SS are observed. The dashed boxes indicate the association of CRISPR-like arrays with copies of the *cagA* genes.

In the genomes of *H. pylori* CHC155, HE93/10_v1, and VN1291, the CRISPR-like arrays identified were found to be integrated into the CagA-PAI (Figure 3). In strain 24-A-EK1, four *cagA* genes associated with four of the six CRISPR-like arrays present in its genome were observed, located approximately 60 kb upstream of the rest of the genes that form the type IV secretion system (T4SS). Additionally, a TnpB endonuclease was located 20 kb upstream of the *cagA* genes (Figure 3d). DRs and spacers associated with complete CagA-PAI exhibit a high conservation among themselves (Table 4), whereas the fragmented CagA-PAI of strain 24-A-EK1 lacked such homology (Table S1).

On the other hand, the CagA-PAI fragment in strain SHIM-010, located around 0.88 Mbp, was marked by the presence of TnpA (transposase) and TnpB (endonuclease) associated with MGEs and lacked adjacent CRISPR-like arrays. This pathogenicity island exhibits characteristics like those of strain 24-A-EK1 (Figure 3d). The T4SS components were located 3 kbp upstream of the *cagA* gene.

### 3.6. EPIYA-Motifs in CagA-PAI Associated with CRISPR-like Arrays

Furthermore, analysis of the genomic context showed that all CagA proteins annotated in the *H. pylori* genomes studied contained repetitive sequences located in the C-terminal region. These sequences, also known as EPIYA motifs (Glutamate-Proline-Isoleucine-Tyrosine-Alanine), are key modulators of host cell proliferation and morphology and are directly related to the pathogenic and oncogenic potential of the strains. Notably, these motifs were present regardless of whether the CagA-PAI was complete or fragmented.

Two scenarios were observed regarding the relationship between EPIYA motifs and CRISPR-like arrays (Table 5). In the first, fragments of CRISPR-like sequences were found integrated into the *cagA* gene, where they are translated into segments containing EPIYA motifs, thus establishing an interesting molecular link. In the second scenario, EPIYA motifs showed no detectable sequential relationship with CRISPR-like sequences. This distinction indicates that the homology observed in the first scenario is sequence-specific and not an artefact of the repetitive structure characteristic of the C-terminal region of CagA. Specific details of the EPIYA motifs integrated into CagA proteins are provided in Figure S1.

## 4. Discussion

The use of multi-bioinformatic tools to identify CRISPR-Cas systems across *H. pylori* genomes revealed that only CRISPRCasFinder detected arrays with evidence level 1 (CRISPR-like arrays), attributable to the CRISPRCasFinder algorithm, which enables their identification. These CRISPR-like arrays were present at high frequency in all strains studied. None of the tools identified a functional CRISPR-Cas system, as these arrays lack the operon structure characteristic of functional systems described in other bacteria [34]. Despite this, the detection of these CRISPR-like sequences allowed exploration of their genomic context and the formulation of hypotheses regarding their potential roles.

The absence of a functional CRISPR-Cas system in *H. pylori* contrasts with the phylogenetically close species *H. mustelae,* which harbours a CRISPR-Cas subtype II-C system [35]. The validation showed that all three bioinformatic tools correctly identified this subsystem, confirming the sensitivity of our approach. Therefore, the CRISPR-like arrays identified on the analysed genomes likely reflect the genomic structure of *H. pylori*. It has been proposed that *H. pylori* may have lost or repurposed its adaptive immunity system to enhance its capacity for genetic adaptation and virulence [12,36]. This scenario is consistent with the marked genomic plasticity characteristic of this species [3]. Although the evolutionary mechanisms underlying this shift remain incompletely understood, the findings presented here support the hypothesis that CRISPR-like arrays in *H. pylori* may have undergone a functional transition toward roles unrelated to adaptive immunity [12,14].

Although possible Cas proteins were identified adjacent to certain CRISPR arrays, these lacked the Cas1-Cas2 complex, which is essential for integrating exogenous DNA fragments into the CRISPR loci [10]. Furthermore, the limitation of DR sequences (>3) and spacers from foreign DNA confirmed the presence of CRISPR-like arrays, consistent with the level 1 evidence of the CRISPRCasFinder tool [16].

The conserved sequences in the CRISPR-like arrays detected in the *H. pylori* genome in Bangpanwimon et al.’s study suggest their potential as a typing method in this species [13]. In this regard, the homology observed in 88% of our CRISPR-like sequences reinforces this structural stability, suggesting that these arrays are not simply random fragments, but rather possess a conserved organisation that could be linked to cellular or adaptive processes not yet described in this pathogen [37]. Particularly, strains HE93/10_v1 and 24-A-EK1 displayed identical spacer sequences at distinct genomic regions. Whilst this pattern resembles self-targeting events described in functional CRISPR-Cas systems [38], in the absence of *cas* genes, it more likely reflects genomic duplication or translocation events facilitated by the high plasticity of the *H. pylori* genome [39].

Notably, this arrangement coexists with the presence of multiple copies of the *cagA* gene, suggesting that these CRISPR-like arrays are integrated at complex genetic loci. This is a similar approach to evolutionary models described for CagA-PAI, where CHA-ud sequences flank the *cagA* gene and are key to the formation of multi-*cagA* genotypes [40,41]. In this sense, the structural association within the CagA-PAI points to a genomic architecture that could be linked to greater pathogenic potential, such as that reported in multi-*cagA* genotypes of hypervirulent Asian strains [40,41]. However, this association requires further investigation.

**Figure 4:**
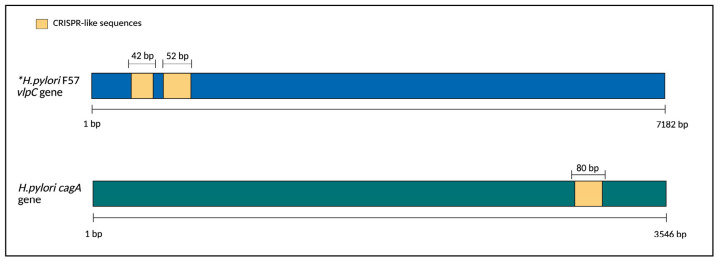
Comparison between the location of CRISPR-like sequences in two different genes associated with *H. pylori* virulence. *, data reported in *H. pylori* F57 (*vlpC*) were adapted from Bangpanwimon et al. [38]. Data corresponding to the *cagA* gene were taken from the results of this study. Figure made in BioRender.com.

The ability to undergo genomic recombination is an essential adaptive feature in *H. pylori* and is clinically relevant [42,43]. Recently, CRISPR-like repetitive sequences were found associated with antibiotic resistance in *H. pylori*, specifically within the *vlpC* gene, homologous to the *vacA* virulence gene, where their presence coincides with mutations that confer metronidazole resistance [14]. The present findings extend these observations by documenting a structural association of CRISPR-like arrays in *cagA* gene duplication and CagA-PAI insertion. In addition, these arrays appear to be integral components of highly plastic genomic regions, reinforcing the idea that these sequences could be important in the genomic plasticity of *H. pylori* (Figure 4).

Analysis of the relationship between CRISPR-like arrays and EPIYA motifs revealed two distinct scenarios (Table 5). Crucially, the absence of this relationship in other cases demonstrates that the observed homology is sequence-specific and not merely an artefact of the repetitive nature of the C-terminal CagA region. This distinction shows a structural association that underscores an architecturally relevant role for these repetitive elements in the *H. pylori* genome [44], while potentially indicating a recombination event between the CRISPR-like repetitive sequences and the C-terminal region of the CagA protein [41,45].

Moreover, the observed link between CRISPR-like arrays and EPIYA motifs is particularly relevant since the conservation of EPIYA motifs, even within fragmented CagA-PAI, indicates selective pressure to maintain CagA functionality, which may explain its persistence even in fragmented pathogenicity islands in *H. pylori* [44].

The structural instability of CagA-PAI in strains SHIM-010 and 24-A-EK1 was observed in the presence of the TnpA and TnpB proteins. These enzymes, described as mediators of gene mobilisation and DNA cleavage [46], were located between the T4SS and *cagA* genes, where the only fragmented pathogenicity islands were identified. The detection of these proteins in conjunction with CRISPR-like arrays highlights a complex genomic mosaic where stable repetitive sequences and elements of active mobilisation coexist. Whilst the direct impact of these endonucleases has not been characterised in *H. pylori*, their ability to induce targeted DNA cleavage in other organisms [47] may suggest regions prone to genomic rearrangement in *H. pylori.* Therefore, this architecture requires future research to explore its true role as a potential regulator of exogenous DNA or as a driver of local genomic instability in these pathogenic bacteria.

In addition, in recent years, prophages have been proposed as a promising strategy for alternative treatments against MDR bacteria [48]. Therefore, the detection of prophages integrated into the genomes of the VN1291, CHC155, SHIM-010, and 29CaP strains analysed is particularly relevant. According to the data obtained using the PHASTEST v3.0, these prophages have an intact structure [29]. To date, all the identified prophages have been described as lysogenic, i.e., they remain latent in *H. pylori* without cytotoxic effects [22,49,50,51]. Furthermore, the characterisation of the different prophages has raised questions about the relationship between bacterial diversity and their presence. Previous findings have shown that prophages integrated into the *H. pylori* genome can act as a vector of horizontal virulence, transcriptionally modulating virulence genes and conferring morphological, motility, viability, and pathogenicity diversification [50,52,53].

A key observation is the genomic proximity of CRISPR-like arrays in the prophages detected in this study. This is especially true of the spatial overlap in strain 29CaP between the prophage Helico 1961P and the CRISPR-like sequences identified in its genome. Notably, 29CaP was the only phage-carrying strain in which the CagA-PAI was absent, a finding of interest given its clinical origin in a patient with gastric cancer [22]. This interaction observed in strain 29CaP could have a compensatory effect that regulates phage activity, such as the retention of virulence-associated genes in the absence of the CagA-PAI.

A different scenario is observed in strain CHC155, where CagA-PAI is present along with prophages. Takeuchi et al. [53] also demonstrated that the KHP30 prophage had a suppressive effect on the expression of the *cagA* gene. Therefore, the coexistence of CagA-PAI, CRISPR-like arrays, and the presence of the KHP30 phage in the strain, also isolated from a patient with gastric cancer [8], could reflect a complex interface for the regulation of this bacteriophage’s activity and its influence on the pathogenicity island. A similar scenario can be inferred for strains SHIM-010 and VN1291, which present equivalent conditions with prophages KHP33 and KHP40, respectively.

However, functional analyses of these CRISPR-like repetitive sequences will be needed to elucidate their role in *H. pylori* and the genomic components described in this study.

## 5. Conclusions

The results of this study reveal that, whilst *H. pylori* lacks a functional CRISPR-Cas system, it possesses a genomic architecture characterised by CRISPR-like repetitive sequences located in regions of high plasticity. Furthermore, the conservation of these sequences, in conjunction with current literature, indicates that these repeats are not merely artefacts, thus giving them greater biological relevance. The structural association of these CRISPR-like arrays with the CagA-PAI, TnpB and TnpA proteins and intact prophages suggests that they could serve as as-yet-unexplained functional or genomic stability elements. Therefore, it is imperative to continue analysing these repetitive sequences through large-scale genomic and phylogenetic studies to determine whether these patterns are conserved across different global lineages. Finally, a deeper functional understanding of this architectural link between CRISPR-like sequences and the *cagA* gene or prophage dynamics could reveal new insights into the adaptive success and pathogenic potential of *H. pylori*.

## Figures and Tables

**Table 1:** Characteristics of the genome collection.

Strain	Accession	Isolation	Condition	Size (Mb)	Study
VN1291	AP026444.1	Vietnam	Gastric cancer	1.7	[8]
CHC155	AP026446.1	Vietnam	Gastric cancer	1.7	[8]
SHIM-010	CP051505.1	Peru	Stomach diseases	1.7	[18]
F57	AP011945.1	Japan	Gastric cancer	1.6	[19]
HE93/10_v1	LT838273.1	Germany	Gastritis	1.7	[20]
24-A-EK1	CP032907.1	Germany	Gastritis	1.7	[21]
29CaP	CP012907.1	Mexico	Gastric cancer	1.6	[22]
7C	CP012905.1	Mexico	Chronic gastritis	1.6	[23]

**Table 2:** CRISPR arrays detected in *H. mustelae* 12198 with CRISPRCasFinder.

Array Type	No. Array	DR Consensus	Identity of DR Sequences	No. Spacers	Location (bp)
CRISPR-Cas subtype II-C system detected	1	GTTTTAGCCACTTCATAAATATGTTTATGCTAAAAT	100%	10	24,706–25,400
Additional CRISPR-like arrays detected	2	ATTGTGATTTTATGTTTTTATCCTTAAACT	100%	1	561,355–561,355
	3	CTATTTTGCGCAGCCTGGGAGGGATTTTG	100%	1	893,577–893,692
	4	TTTTCTCCTCCTGTGTGTTTGGAGTGCTAGCTGGGGTGGTG	100%	1	919,307–919,437
	5	TTGGCTCCTAGCGTGATGGTGTTAGT	100%	1	972,985–973,091
	6	CCCCCACCCCGGTCAGGCTCTAC	95.65%	1	988,649–988,747
	7	AAGCCCTAGATGAAGAGAAAAGATATCAAGCACAA	100%	1	1,533,491–1,533,604

**Table 3:** Intact prophages identified in *H. pylori* genomes using PHASTEST v.3.

Strain	Sequence	Size (kb)	Location (bp)	Closest Phage	Attachment Site	Completeness Score
VN1291	1	28	757,548–786,311	Phage Helico KHP40 (NC_019931)	Yes	140 (intact)
CHC155	1	34	756,907–756,907	Phage Helico KHP30 (NC_019928)	Yes	145 (intact)
SHIM-010	1	40	179,744–219,789	Phage Helico KHP33 (NC_016568)	Yes	110 (intact)
29CaP	1	44	1,330,396–1,374,469	Phage Helico 1961P (NC_019512)	Yes	145 (intact)

**Table 4:** CRISPR-like arrays associated with complete CagA-PAI.

Strain	CRISPR Array No.	Sequence
VN1291	2	**GAAGAGCCCATTTATGCTCAAGTT** *AATAAAAAGAAAACAGGACAAGTAGCTAGCCCT* **GAAGAGCCCATTTACGCTCAAGTT**
CHC155	2	**GAAGAGCCCATTTA** **CGCTCAAGT** *CAATAAAAAGAAATCAGGACAAGCAGCTAGCCCT* GAAGAGCCCATTTACGCTCAAGT*
HE93/10_v1	1	GAGAA*CCCATTTATGCTCAAGTT *AATAAAAAGAAAACAGGACAAGTAGCTAGCCCT* **GAAGAACCCATTTATGCTCAAGTT**

The sequence structure is organised as follows: **DR**, *spacer*, **DR** (bold/italic formating style, respectively); *, deletion in the alignment; in red, nucleotide substitution.

**Table 5:** EPIYA-Motifs identified and associated with CRISPR-like arrays.

Case	Strain	EPIYA Type	CagA-PAI Type	CRISPR-like Array No. Associated	EPIYA Type Associated
Relationship between CRISPR-like arrays—EPIYA motifs	CHC155	A, B, C	Complete	2	A, B
	HE93/10_v1	A, B, C	Complete	1–3	A, B
	VN1291	A, B, C	Complete	2	A, B
	24-A-EK1	Variant A, B, C	Fragmented	1–4	Variant A, C
EPIYA motifs unrelated to CRISPR-like arrays	SHIM-010	A	Fragmented	None	None
	F57	A, B, C	Complete	None	None

EPIYA motifs are classified into types A, B, and C based on the specific amino acid sequences flanking the phosphorylation site. Variants are sequences containing amino acid substitutions that differ from the canonical consensus but maintain the EPIYA architecture [33].

## Data Availability

Data is contained within the article or Supplementary Material.

## Supplementary Materials

The following supporting information can be downloaded at https://www.mdpi.com/article/10.3390/pathogens15050461/s1. Data is contained within the article or supplementary material. Figure S1. Classification of EPIYA-Motifs detected in *cagA* genes of *H. pylori*. Table S1. CRISPR-like arrays identified in *H. pylori* with CRISPRCasFinder and characteristic patterns in Direct Repeats and Spacers. Table S2. Cas proteins detected with CRISPRCasFinder in *H. mustelae*. Table S3. CRISPR-Cas system in *H. mustelae* detected with CRISPRCasFinder. Table S4. Associated and neighbor proteins of CRISPR arrays detected. Table S5. General characteristics of phage detected in *H. pylori* with PHASTEST v.3.

## Abbreviations

The following abbreviations are used in this manuscript:

*cagA*	Cytotoxin-associated gene A
CagA-PAI	Islands of Pathogenicity Associated with the CagA Cytotoxin
Cas	CRISPR-associated protein
CRISPR	Clustered Regularly Interspaced Short Palindromic Repeats
DR	Direct Repeats
HGT	Horizontal Gene Transfer
MGEs	Mobile Genetic Elements
