# Supplementary material for: New Insights into CRISPR-like Arrays in Helicobacter pylori: An Exploratory Analysis from Genomic Data
Source: Pathogens. 2026 Apr 24;15(5):461. doi: 10.3390/pathogens15050461 (PMC13209394; doi:10.3390/pathogens15050461)
Supplement: Supplementary file 1 [file pathogens-15-00461-s001.zip › Supplementary_figures.pdf]

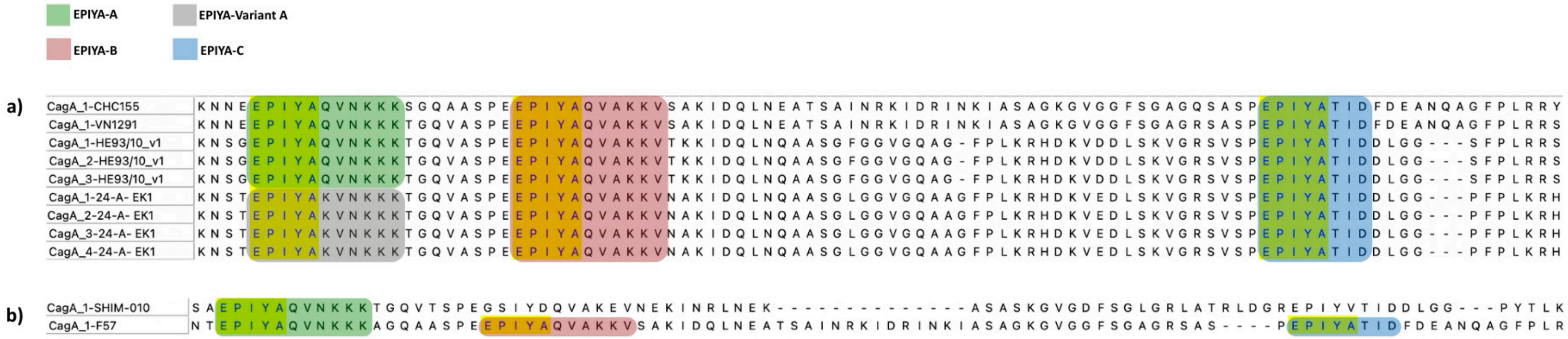

Figure S1. Classification of EPIYA-Motifs detected in *cagA* genes of *H. pylori*. a) Strain relationship between CRISPR-like sequence – EPIYA motifs; b) Strain unrelated EPIYA to CRISPR-like sequences.
